# The Bergen 4-Day Treatment for panic disorder patients in a rural clinical setting: a long-term follow-up study

**DOI:** 10.1186/s12888-024-06445-0

**Published:** 2025-01-02

**Authors:** Thorstein Olsen Eide, Thorbjørn Olsen, Hans Hansen, Bjarne Hansen, Stian Solem, Kristen Hagen

**Affiliations:** 1 Department of Psychiatry, Molde Hospital, Møre og Romsdal Hospital Trust, Molde, Norway; 2 Center for Crisis Psychology, Faculty of Psychology, University of Bergen, Bergen, Norway; 3 Bergen Center for Brain Plasticity, Haukeland University Hospital, Bergen, Norway; 4 Førde Hospital, Førde Hospital Trust, Førde, Norway; 5 Division of Psychiatry, Sørlandet Hospital, Kristiansand, Norway; 6 Department of Psychology, Norwegian University of Science and Technology, Trondheim, Norway; 7 Department of Mental Health, Norwegian University of Science and Technology, Trondheim, Norway

**Keywords:** Panic disorder, Cognitive behavioural therapy, Bergen 4-Day treatment, Long-term effectiveness, Rural clinical setting

## Abstract

**Background:**

The Bergen 4-Day Treatment (B4DT) is a concentrated cognitive behaviour therapy (CBT) approach that has shown promise in treating panic disorder (PD). However, the effectiveness of the B4DT, particularly regarding long-term outcomes in rural clinical settings, remains underexplored.

**Methods:**

A total of 58 patients were included using a naturalistic open-label trial design. Patients were assessed at 12-month follow-up. Measures included the Panic Disorder Severity Scale (PDSS), the Patient Health Questionnaire-9 (PHQ-9), and the Generalized Anxiety Disorder-7 scale (GAD-7).

**Results:**

The study revealed significant and lasting reductions in PD symptoms, with a high rate of remission maintained at 12-month follow-up (82.8%). Regarding the secondary outcomes, significant improvements in symptoms of depression and generalized anxiety were also shown.

**Conclusions:**

The B4DT represents a promising treatment approach for PD, demonstrating stable long-term outcomes in rural settings. This finding supports the potential of concentrated CBT formats in achieving sustained symptom improvement in patients with PD, warranting further investigation and broader implementation.

**Trial registration:**

The study was reviewed by the Regional Committee for Medical Research Ethics Northern Norway, REK North (REK Nord2021/273145).

## Introduction

Panic disorder (PD) is an anxiety disorder characterized by the occurrence of recurrent, unanticipated panic attacks. These panic attacks manifest as sudden and intense episodes of anxiety, often accompanied by physiological symptoms such as a rapid heart rate, hyperventilation, sweating, trembling, and dizziness. Following panic attacks, individuals typically experience either a substantial maladaptive alteration in their behaviour or an excessive preoccupation with the anticipation of future panic attacks and their potential consequences [1]. PD has been demonstrated to have a significant negative impact on quality of life [2] and on work and general functioning, especially with a comorbid diagnosis of agoraphobia [3]. The lifetime prevalence of PD worldwide has been estimated to be 1.7%, with a higher prevalence reported in Western Europe and North America [4]. 80% of patients with PD are estimated to have lifetime comorbid mental disorders, with the most common disorders being other anxiety disorders and mood disorders [4].

Cognitive behavioural therapy (CBT) has been extensively researched and shown to be an effective treatment format for PD patients with or without agoraphobia [5, 6]. A recent network meta-analysis revealed that when studies with a high risk of bias were excluded, CBT emerged as the sole psychotherapy that was more effective than treatment as usual (TAU) for PD [6]. Pharmacological treatment, specifically treatment with serotonin reuptake inhibitors (SSRIs), is another effective treatment option for patients with PD [7]. CBT for PD has been demonstrated to be effective in a variety of settings including private practise [8]. It has been demonstrated that CBT for PD with agoraphobia can be intensified without affecting therapy outcome [9], and a recent meta-analysis revealed no differences in treatment effects between regular CBT and intensive CBT for PD patients with or without agoraphobia [10]. Intensive CBT programs might increase access to therapy, prompt faster return to work, lower drop-out rates, and increase quality of life [9, 11].

The Bergen 4-Day treatment (B4DT) is a form of concentrated CBT treatment that has demonstrated promising results in the treatment of obsessive-compulsive disorder (OCD) [12–18] and social anxiety disorder [19]. The B4DT demonstrated promising results for PD. In a pilot study including 30 patients, 72% of patients were in remission at three month follow-up [20], in a replication study including 30 patients in a new clinical setting 86.7% of patients were in remission at three month follow-up [21], in a hybrid video and face-to-face treatment of 50 patients during the COVID-19 pandemic 62% were classified as in remission at three month follow-up [22], and a study including a larger sample of 58 patients in a rural clinical setting were 81% of the patients were in remission at three month follow-up [23]. Only one study examining the long-term effectiveness of the B4DT for PD, which indicated that the treatment results were stable at long-term follow-up, has been conducted [24]. It is important to examine the long-term outcomes of the B4DT for PD, as there is a lack of long-term follow-up data on CBT for patients with PD in general [25].

The aim of the present study was to evaluate the long-term effectiveness of the B4DT for patients included in the study by Eide et al. [23], who examined a sample of 58 patients receiving the treatment as part of ordinary care in a rural clinical setting. Studying patients from rural areas may be of interest as they are often less represented in clinical trials, may have other sociodemographic characteristics, have longer delays before seeking treatment, and usually have poorer access to mental health services [26, 27].

Due to differences in their goals and designs, efficacy and effectiveness studies have different strengths and weaknesses. The goal of an efficacy study is to examine interventions under highly controlled conditions, which strengthens internal validity. On the other hand, effectiveness studies aim to examine treatment under conditions that mirror real-world conditions, decreasing internal validity but increasing external validity [28]. We hypothesized that the long-term outcomes of the B4DT would be associated with high remission rates, significant and large reductions in the primary PD outcome measures, and a significant decrease in the secondary outcome measures of depression and generalized anxiety. Based on previous studies of the B4DT, we hypothesized that the long-term outcomes would show high remission rates (about 80%).

## Methods

### Participants and procedure

This was a naturalistic open-label effectiveness study examining the long-term (12-month follow-up) outcomes of 58 patients receiving the B4DT as a part of ordinary clinical care.Patients were consecutively recruited from a standard outpatient clinic within the public mental health care system in Norway. Treatment took place from 2017 to 2020 [23]. The study was conducted at a standard outpatient clinic within the public mental health care system in one of the most rural and sparsely populated areas of Norway. The clinic serves a catchment area of approximately 35,000 people spread over a large geographical region, with patients potentially traveling several hours to reach the clinic. Patients were referred to receive the B4DT either by their general practitioners or other clinicians in the public mental health care system. Patients were diagnosed pretreatment by an experienced clinician using the Mini International Neuropsychiatric interview (M.I.N.I) [29]. Patients who med the diagnostic criteria for PD with or without agoraphobia were offered treatment. Patients were excluded if they had psychosis, were suicidal or in an unstable phase of bipolar disorder, had active substance abuse, or were not fluent in Norwegian.

The Regional Committee for Medical Research Ethics in Northern Norway (REK North, reference REK Nord-2021/273145) reviewed the study. All participants provided informed written consent, and were informed that they could withdraw from the study at any time.

Patients were offered a long-term follow-up session 12 months after completing the B4DT, during which possible difficulties or successes encountered after the treatment could be addressed. Patients were asked to complete self-report questionnaires online before or during the long-term follow-up session. During the long-term follow-up session, the Panic Disorder Severity Scale (PDSS) was also administered. A total of 96.5% (*n* = 56) of the original sample attended the follow-up session.

The demographic pretreatment information of the sample can be found in Table 1. Most patients had PD and agoraphobia (86.6%, *n* = 52). The majority of the sample was female (74%, *n* = 43), 74.1% (*n* = 43) of the sample had at least one comorbid disorder, and 53.4% (*n* = 31) of the sample had previously received treatment for PD. Regarding medication use, 44.8% (*n* = 26) of the sample had used SSRIs, and sleep medication usage was self-reported by 29.3% of the patients. All patients using medication were instructed to maintain the same medication types and dosages before and during the treatment period. Detailed dosage information was not collected. Patients using benzodiazepines (8.5%, *n* = 5) were encouraged to avoid their use during and directly after exposure sessions. All patients adhered to this instruction, and none reported withdrawal effects during the treatment period.

### Assessment

The primary outcome measure was, as in the original study, the PDSS score [30]. The PDSS is a 7-item interview assessing the severity of PD symptoms. The items are rated on a four-point Likert-type scale, and the total score ranges from 0 to 28 points, with higher scores indicating greater severity of PD symptoms. The interview has previously demonstrated good reliability and validity and is sensitive to change [30]. The Panic Disorder Severity Scale (PDSS) assessment before treatment was conducted by one of the therapists involved in the treatment. Post-treatment assessments at 3 months and 12 months follow-up were conducted by an independent therapist not involved in the treatment.

The secondary outcome measures were self-reported depression and generalized anxiety questionnaire scores. The Patient Health Questionnaire-9 (PHQ-9) [31] is a brief questionnaire based on the DSM criteria for depression. The nine items are rated on a four-point Likert-type scale ranging from 0 (not at all) to 3 (almost every day). Total scores range from 0 to 27 points, with higher scores indicating greater severity of depression. A PHQ-9 score from 0 to 4 points indicates the absence of depression, a score of 5–9 points indicates mild symptoms, a score of 10–14 points indicates moderate symptoms, a score of 15–20 points indicates moderate/severe symptoms, and a score of 20 + points indicates severe symptoms. The questionnaire has demonstrated high sensitivity and specificity for detecting depression and good validity, reliability, and sensitivity to change [31, 32]. The PHQ may differ from other and more comprehensive measures of depression such as the BDI-II, but a psychometric comparison of the two concluded that using the PHQ-9 may be more advantageous [33].

The Generalized Anxiety Disorder-7 (GAD-7) [34] is a brief seven-item questionnaire that was developed to measure symptoms of generalized anxiety. The items are rated on a four-point Likert-type scale ranging from “0” to “3”, and total scores range from 0 to 21 points, with higher scores indicating greater symptom severity. A total score greater than 10 points is considered to indicate clinically severe generalized anxiety. The GAD-7 has demonstrated good psychometric properties [32, 34] and is also an appropriate measure of anxiety symptoms [35]. Psychometric comparisons between the GAD-7 and the more exhaustive Penn State Worry Questionnaire, suggested that the GAD-7 was more strongly recommended for clinical research [36].

### Treatment

The B4DT for PD is a four-day protocol with a three-month follow-up booster session. The B4DT treatment for PD involved both in vivo exposure to external situations that patients tended to avoid or feared significantly, and interoceptive exposure to bodily sensations that elicited fear or had been previously avoided. Patients were encouraged to gain experience in a diversity of relevant settings. Typical exposure tasks included physical exercises and hyperventilation to increase physiological arousal, as well as exposure to agoraphobic situations such as shopping malls, buses, and other environments based on each patient’s specific avoidance patterns and fears.

The focus of all exposure tasks was practicing the Leaning-In Technique (LET), where patients actively attempted to elicit anxiety instead of avoiding it or trying to regulate it. This approach is presented to the patients as Intention-Focused Cognitive Behavioral Therapy (IF-CBT), which posits that discomfort in anxiety disorders largely results from the decisions patients make after perceiving a situation as potentially dangerous—their “intention” or “project.” According to IF-CBT, the intention to prevent or avoid feared events leads to emotional activation and discomfort, reinforcing the assumption of danger and amplifying future reactions.

In practicing LET, patients learned to identify moments when they were tempted to initiate avoidance or safety behaviors and then chose to behave as the situation or internal trigger was not dangerous by “leaning in.” Instead of focusing on disconfirming catastrophic thoughts or reducing anxiety during exposure, the treatment aimed to find anxiety-eliciting situations or thoughts that could be practiced using the LET technique. The therapy emphasized that small tasks performed with the correct intention were more effective than longer or more anxiety provoking tasks without a focus on recognizing and altering the patient’s underlying intentions.

By shifting their intention from avoidance to actively engaging with feared stimuli, patients worked toward altering their internal responses and reducing the discomfort associated with PD. This method fosters a proactive stance against anxiety, encouraging patients to confront their fears directly and change the patterns that maintain their anxiety over time.

The Bergen 4-Day Treatment (B4DT) is an intensive, in-person treatment delivered individually within a group setting. Each group consisted of 3 to 6 patients and an equal number of therapists, ensuring personalized attention. Over the study period, several groups were conducted. The treatment was administered by a team of five certified 4-day treatment therapists. Certification required participation in at least three treatment groups with hands-on supervision by an experienced 4-day therapist. Additionally, the group leaders were certified, having participated in at least six groups and received certification. The team for this study included two psychologists, two social workers, and one psychiatric nurse, and their experience in treatment of PD ranged from six months to 30 years.

Day one involves three hours of psychoeducation on panic disorder, treatment introduction, and exposure task preparation in a group setting. The subsequent two days, each lasting seven hours, focuses on interoceptive and in vivo exposure tasks, emphasizing personalized experiences and sharing experiences in a group setting. The final day, which lasts three hours, centres on maintaining treatment effects and planning exposure activities for the following three weeks, during which patients self-monitor without receiving therapist feedback. A booster session three months posttreatment helps consolidate gains without further exposure exercises.

### Statistical analysis

There was a low degree of missing data in the present study. All patients reported symptoms using the PDSS at the pretreatment session. At the posttreatment, three-month follow-up, and long-term follow-up sessions, 8.6% (*n* = 5), 6.8% (*n* = 4) and 3.4% (*n* = 2) of the patients had missing PDSS data, respectively. For the self-report questionnaires, 11.2% of the patients were missing data across all the measurement points. To perform the statistical analysis, we used IBM SPSS statistics (Version 29). The expectation maximization (EM) method within SPSS version 29 was utilized to impute missing data. This method is considered reliable when the missing data constitute less than 25%, when the missing data are random, as confirmed by Little’s missing completely at random (MCAR) test χ² (27) = 29.471, *p* = .338, and when the data demonstrate a normal distribution, as indicated by the Kolmogorov‒Smirnov test [37]. All conditions were met in the present study.

The use of the EM method allowed the use of repeated-measure ANOVAs in the present study to examine changes in symptoms from pretreatment to posttreatment, at the three-month follow-up, and at the 12-month follow-up. If Mauchly’s test of sphericity was significant, Greenhouse–Geisser correction was used. For post hoc analysis, Bonferroni correction was used.

The criteria for treatment response and PD remission were defined based on the consensus criteria established by Furukawa et al. [38]: patients with PD only were considered to be in remission if their scores were 5 points or less, while those with PD and agoraphobia were considered to be in remission if their scores were 7 points or less.

## Results

The patients showed a significant reduction in PD symptoms over time, as measured by the PDSS (shown in Table 2). The results of the repeated-measures ANOVA demonstrated a significant reduction in PD symptoms over time (*F*(2.482, 141.452) = 197.56, *p* < .001, partial *µ*^2^ = 0.776). There was a significant reduction in PD symptoms from pretreatment to the 12-month follow-up (*d =* 3.11, *p* < .001), and we found no significant change in PD symptoms from the 3-month follow-up to the 12-month follow-up (*d* = 0.084, *p* = 1.00). There were no dropouts from the treatment.

For symptoms of generalized anxiety, there was a significant reduction over time (*F*(2.550, 145.351) = 44.275, *p* < .001, partial *µ*^2^ = 0.437). There was a significant reduction in symptoms of generalized anxiety from pretreatment to the 12-month follow-up (*d* = 1.30, *p* < .001). We found no significant change in symptoms of generalized anxiety from the 3-month follow-up to the 12-month follow-up (*d* = 0.17, *p* = 1.00). A score of 10 or more on the GAD-7 is recommended as a cut-off point for of generalized anxiety disorder [34]. A total of 72% were above cut-off at pretreatment compared to 19% at 12-month follow-up.

For depression, there was a significant reduction over time (*F*(2.177, 124.085) = 32.23, *p* < .001, partial *µ*^2^ = 0.361). Symptoms of depression significantly decreased from pretreatment to the 12-month follow-up (*d* = 0.86, *p* < .001). There were no significant changes in symptoms of depression from the 3-month follow-up to the 12-month follow-up (*d* = 0.04, *p* = 1.00).

A score of 10 or more is the most common cut-off point for depression on the PHQ-9 [39]. At pretreatment 67% scores above cutoff compared to 24% at 12-month follow-up.

At the long-term follow-up, following the Furukawa criteria, 82.8% of the participants were classified as being in remission (see Table 3). A total of 75.9% of the participants were classified as having symptoms that were “very much improved”, 10.3% were classified as having symptoms that were “much improved”, and 10.4% were classified as having “no improvement” or “minimal improvement” in their symptoms.

## Discussion

This is the second study investigating the long-term efficacy of the B4DT for PD patients. The initial study on the long-term outcomes of the B4DT for PD by Eide et al. [24] demonstrated that the treatment outcomes remained consistent for 30 patients. The findings of our study further strengthen the finding that treatment effects observed at the 3-month follow-up remain stable in the long term. Furthermore, this study extended the findings by including a larger sample from a rural setting. We observed no significant difference in PD symptoms between the 3-month follow-up and the 12-month follow-up, and there were no significant changes in the secondary outcome measures for depression or generalized anxiety from the three-month follow-up to the twelve-month follow-up. This is in line with previous findings of the B4DT for OCD [13, 14, 16, 18], which indicate that the effects of intensive treatment are stable across time.

Compared to the first long-term follow-up study on the B4DT by Eide et al., [24] there were no significant differences in PDSS scores at the long-term follow-up (*p* = .46, *d* = 0.13). Both studies found large treatment effects, but the effect size was larger in the study by Eide et al. [24] (*d* = 5.03) than in the current study (*d* = 3.11). This is likely a result of the first study having a significantly higher pretreatment PDSS score.

Although several studies have demonstrated that intensive or concentrated CBT is a promising and acceptable treatment format for patients with anxiety disorders, including PD patients [10, 11, 40, 41], few studies have examined the long-term outcomes of this treatment. Some studies have examined the long-term outcomes of intensive/concentrated CBT for PD patients, and the findings align and indicate large effect sizes [40]. A recent meta-analysis including both randomized controlled trials and open-label studies revealed no difference in remission status or effect size for patients with PD between effectiveness and efficacy studies [42]. The patients in the current study had a greater remission rate (82.8%) than did the patients in the effectiveness and efficacy studies included in the meta-analysis (65.6–66.3% for PD patients and 57.0-63.8% for agoraphobia patients). Although comparisons of the results from one study to those of a meta-analysis should be performed with caution, these findings indicate that the B4DT is a promising treatment for patients with PD. The positive changes observed after treatment resembles positive effects found in a related study of a 1-week behavioural treatment for agoraphobia, suggesting that panic disorder treatment can be intensified and allow for more rapid return to work and daily activities [9].

The present study was a naturalistic open-label effectiveness study, and the goal of these types of studies is to examine a treatment under conditions as close as possible to real-world conditions. This means that this study has several important limitations and strengths. The present study had no control conditions. There was no randomization, and there was no collection of data on whether the patients in the sample had received any other form of psychotherapy or started/changed psychotropic medication during the long-term follow-up period. A limitation of our study is that we did not control for when patients started their medication or the specific dosages used, as these were managed by their general practitioners or psychiatrists. We cannot exclude the possibility that medication changes may have contributed to the observed improvements. A further limitation of our study is that we have no data on the proportion of patients who were offered the intensive program but declined participation. This lack of information on acceptance rates may affect the generalizability of our findings and has implications for the external validity of the study.A strength of the present study is that the treatment was given as part of ordinary care at a small clinic in a rural setting, contributing to the high external validity of the results. We consider the sample representative because patients were consecutively included from routine clinical practice, and the study employed minimal exclusion criteria, enhancing the external validity of our findings. There was also a low degree of missing data, with a 96.4% response rate on the PDSS at the long-term follow-up session. The promising results suggest that the training and implementation procedures developed for the B4DT have been successful. Also at the current clinic as some of the therapist had long experience in the treatment of PD and also participated in precious research on CBT for PD [43].

The present study demonstrated that B4DT results obtained at the three-month follow-up were stable in the long term. This indicates that brief treatment could be associated with lasting changes. The treatment was conducted in a rural clinical setting with limited resources, suggesting that the treatment can be effectively implemented in rural clinical settings.

**Table 1:** Demographic information (pretreatment)

Variable	M(SD)/*N*(%)
Age	33.6 (12.0)
Female sex	43 (74.1%)
Duration (in years) of the disorder	9.5 (10.3)
Previous treatment	31(53.4%)
With agoraphobia	52 (89.6%)
Comorbidity (any disorder)	43 (74.1%)
Depression	23 (39.7%)
Social anxiety disorder	19 (32.8%)
GAD	19 (32.8%)
PTSD	9 (13.8%)
Psychotropic medication use	
SSRI	26 (44.8%)
Sleep medication	17 (29.3%)

Note. GAD; Generalized anxiety disorder; PTSD, Posttraumatic stress disorder; SSRIs, Selective serotonin reuptake inhibitors

**Table 2:** Results (*M* and *SD*) for the primary and secondary outcome measures (*n* = 58)

Variable	Pre	Post	3-m follow-up	12-m follow-up	d = Pretreatment–12-m follow-up
PDSS	16.10 (3.90)	4.72 (2.77)	2.81 (3.38)	3.14 (4.40)	3.11
GAD-7	12.31 (4.09)	6.31 (4.31)	5.50 (4.05)	6.31 (5.05)	1.30
PHQ-9	12.59 (5.87)	6.77 (4.98)	6.95 (5.40)	7.20 (6.60)	0.86

Note. PDSS, Panic Disorder Severity Scale; GAD-7, Generalized Anxiety Disorder-7 scale; PHQ-9, Patient Health Questionnaire-9. d = Cohen’s *d = (M*_*pre*_*– M*_*post*_*)/SD*_*pooled*_

**Table 3:** Status at follow-up based on the criteria of Furukawa et al

	Posttreatment, *N* (%)	3-m follow-up, *N* (%)	12-m follow-up, *N* (%)
Very much improved (75–100%)	27 (46.6%)	39 (67.2%)	44 (75.9%)
Much improved (40–74%)	23 (39.7%)	11 (19.0%)	6 (10.3%)
Minimally improved (10–39%)	1 (1.7%)	3 (5.2%)	3 (5.2%)
No improvement (0–10%)	2 (3.4%)	1 (1.7%)	3 (5.2)
Missing data	5 (8.6%)	4 (6.9%)	2 (3.4%)
Remission	42 (72.4.%)	47 (81.0%)	48 (82.8%)

Note. Improvement categories are based on improvement in scores on the Panic Disorder Severity Scale

## Data Availability

The data that support the findings of this study are not openly available due to reasons of sensitivity and are available from the corresponding author upon reasonable request. The data are stored in a controlled access database at Haukeland University Hospital.

## Abbreviations

B4DT: Bergen 4-Day Treatment
CBT: Cognitive Behaviour Therapy
GAD: Generalized Anxiety Disorder
PD: Panic Disorder
PDSS: Panic Disorder Severity Scale
PHQ-9: Patient Health Questionnaire-9
PTSD: Post- Traumatic Stress Disorder
GAD-7: Generalized Anxiety Disorder-7 scale
SSRIs: Selective Serotonin Reuptake Inhibitors
LET: Leaning in Technique
MCAR: Missing Completely At Random
EM: Expectation Maximization
ANOVAs: Analysis of Variance
DSM: Diagnostic and Statistical Manual of Mental Disorders
